# Aberrant coronary artery spasms cause ST‐T segment depression during endovascular ablation of atrial flutter

**DOI:** 10.1002/ccr3.1023

**Published:** 2017-06-21

**Authors:** Eva A. H. Lanters, Adriaan Coenen, Marisa M. Lubbers, Koen Nieman, Natasja M. S. de Groot

**Affiliations:** ^1^ Department of Cardiology Erasmus Medical Center Rotterdam Rotterdam The Netherlands; ^2^ Department of Radiology Erasmus Medical Center Rotterdam Rotterdam The Netherlands

**Keywords:** Aberrant coronary artery, cavotricuspid isthmus ablation, coronary anomaly, coronary artery spasm

## Abstract

ST‐T segment depression during right‐sided endovascular catheter ablation is not only caused by obstructive coronary artery disease or (induced) tachycardia. Clinicians should also consider coronary artery spasms evoked by manipulation of nearby catheters, especially in patients with abnormal coronary artery anatomy.

## Introduction

ST‐T segment deviation observed during electrophysiology studies can be the result of, for example, obstructive coronary artery disease or (induced) tachycardia. However, in some cases, ST‐T segment abnormalities are unexpectedly caused by spasms of the coronary arteries, induced by mechanical manipulation of endocardial catheters. This phenomenon has mainly been described during *left*‐sided ablation procedures. ST‐T segment elevation due to coronary artery spasm has been reported during ablation of a left lateral accessory pathway 1 or during isolation of the right inferior pulmonary vein 2. We report a case of ST‐T segment deviation during a *right*‐sided endovascular ablation in a patient with uncommon coronary artery anatomy.

## Case Report

**Figure 1:**
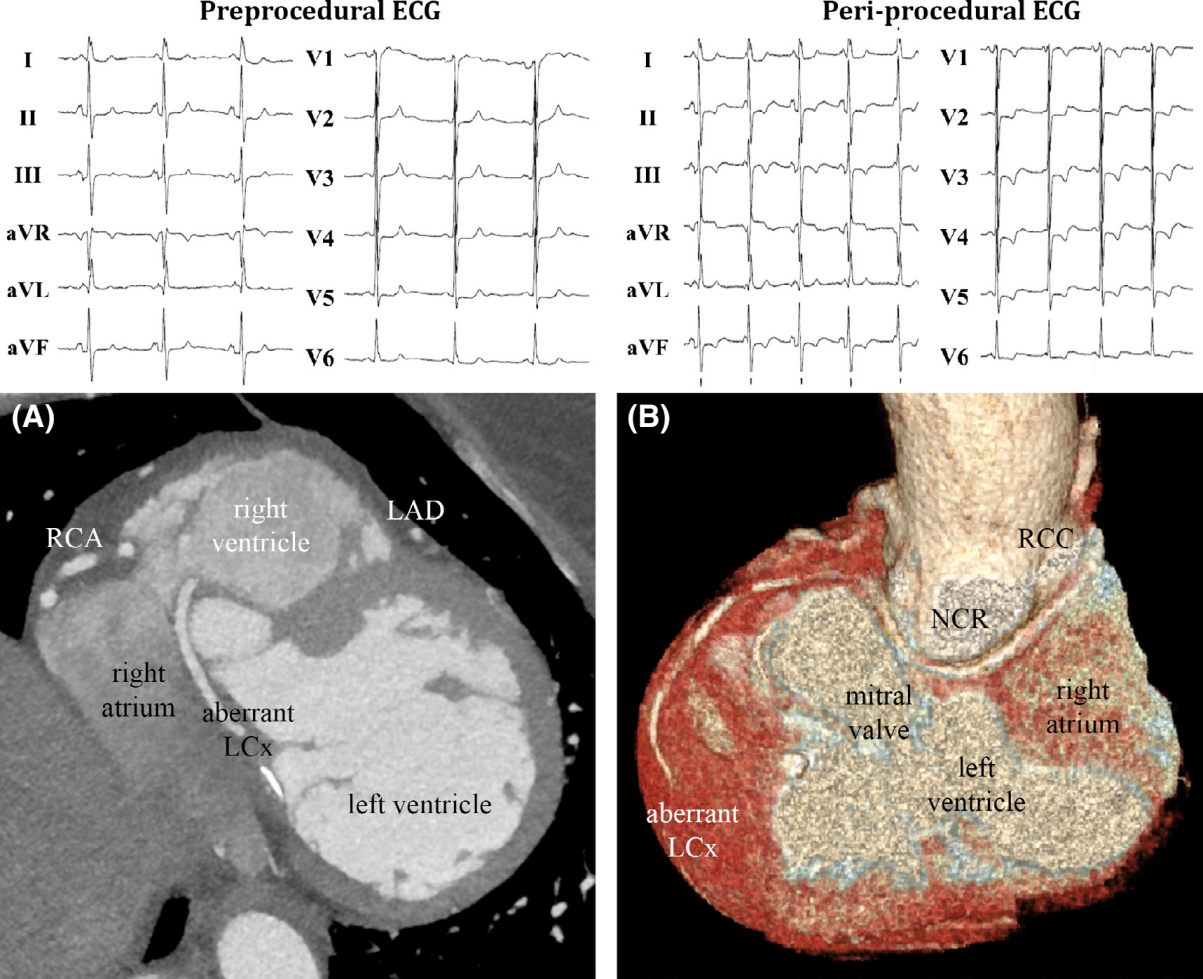
ECG abnormalities caused by coronary anomaly. Surface electrocardiogram at baseline (upper left panel) and during ST‐T segment deviation (upper right panel). Lower panels: CT reconstructions of the aberrant LCX originating from the right coronary cusp. (A) CTA showing the close relation/distance between the aberrant LCX and the right atrium. Catheters were in very close proximity (<2 mm) of the LCX. (B) 3D reconstruction form the CTA showing the course of the aberrant LCX from the right coronary cusp, close to the tricuspid valve, right atrium, mitral valve left atrium, and the lateral left ventricle wall. RCC, right coronary cusp; NCR, noncoronary cusp; LCX, left circumflex coronary artery; LAD, left anterior descending; RCA, right coronary artery.

A 63‐year‐old woman was referred for catheter ablation of a counterclockwise atrial flutter. She experienced self‐limiting palpitations without concomitant complaints. She had a good exercise tolerance without angina or dyspnea. Her medical history included mitral valve repair for prolapse of the posterior leaflet seven years prior to ablation. Coronary angiogram at that time showed no significant coronary artery disease, yet an abnormal origin of the left circumflex artery (LCX) was observed. Echocardiographic evaluation demonstrated that the left ventricular function was mildly impaired, due to an aneurysmatic inferolateral wall. Left atrial dimension was 49 mm. The ablation procedure was performed under local anesthesia. As demonstrated in the upper left panel of Figure 1, the baseline ECG showed sinus rhythm (cycle length 1204 ms) without ST‐T segment depressions. After the introduction of the catheters into the right atrium, transient sinus bradycardia (40 beats/min) and ST segment depression with T‐wave inversion (upper right panel) developed at the precordial leads, without angina or dyspnea. Transthoracic echocardiographic examination showed no abnormalities. To confirm the involvement of the cavotricuspid isthmus in the atrial flutter reentrant circuit, induction of the arrhythmia was attempted. Isuprel was injected to facilitate inducibility. During the administration of isuprel, due to initial noninducibility of any arrhythmia, the transient (asymptomatic) ST‐T segment deviations recurred. At the end of the otherwise successfully performed ablation of the cavotricuspid isthmus, the ECG was normalized. Postprocedural continuous rhythm monitoring showed no recurrences of the ST‐T segment depression. Hence, the patient was discharged from the hospital.

The patient was scheduled for coronary CT angiography for the evaluation of the coronary arteries. A mild (25–50% lumen narrowing) calcified stenosis was seen in the mid‐left anterior descending artery. However, the previously described congenital anomaly was observed, as demonstrated in the right panels of Figure 1. The LCX originates from the right coronary cusp and runs dorsal of the ascending aorta, ventral to the left atrium. This aberrant LCX runs very close (<2 mm) to the caudal junction of the inferior caval vein and right atrium. The sinus node artery also originates from the aberrant LCX. The inferior caval vein also has an uncommon pathway, reaching the right atrium from a more cranial position. Hence, it is likely that in this specific patient, peri‐procedural catheter manipulation in the proximity of the aberrant LCX provoked spasms, which in turn caused transient subendocardial ischemia. This would explain both the ST‐T segment deviations and sinus bradycardia in the absence of significant coronary artery stenosis.

## Discussion

Coronary anomalies are observed in approximately 1.3% of the patients undergoing coronary angiography and are usually benign 3. The vast majority of these patients (87%) has anomalies in the origin of the coronary artery or its distribution 3. Spasms of a coronary artery cause strong vasoconstriction, resulting in a transient (sub)total occlusion of the vessel. When present in the context of Prinzmetal or variant angina, recurrent episodes occur predominantly at rest, but can also be provoked by exercise 4. Catheter‐induced spasms of a coronary artery during coronary angiography occur in 2.9% of the cases 5. The onset of a spasm as a result of left‐sided endocardial ablative procedures has been described in several case reports. However, up until now there was only one case presenting a coronary artery spasm during ablation of a right lateral accessory pathway in a pediatric patient with normal coronary anatomy 6. In this case, we believe that close proximity manipulation of endocardial catheters during a right‐sided atrial flutter ablation induced spasms of an aberrant LCX.

## Conclusion

ST‐T segment depression and sinus bradycardia during atrial flutter ablation in a 63‐year‐old woman can result from catheter‐induced aberrant LCX spasms.

## Conflict of interests

None.

## Authorship

EL: helped in drafting the manuscript, data acquisition, and final approval. AC: contributed to drafting the manuscript, data acquisition, and final approval. ML: critically revised the manuscript, and helped in data design, and final approval. KN: critically revised the manuscript, and contributed to data interpretation, and final approval. NdG: critically revised the manuscript, and helped in data interpretation, and final approval.
